# Assessment of Cattle Owners’ Knowledge, Attitudes and Practices Regarding Bovine Tuberculosis in the Eastern Cape Province, South Africa

**DOI:** 10.1002/puh2.70313

**Published:** 2026-07-22

**Authors:** Effort Maguta, Anita Michel, Ishmael Festus Jaja

**Affiliations:** ^1^ Department of Livestock and Pasture Science Faculty of Science and Agriculture University of Fort Hare Alice South Africa; ^2^ Department of Veterinary Tropical Diseases Faculty of Veterinary Science University of Pretoria Onderstepoort South Africa; ^3^ Department of Agriculture and Animal Health School of Agriculture and Life Sciences University of South Africa Roodepoort South Africa

**Keywords:** *Mycobacterium bovis*, *Mycobacterium tuberculosis*, One Health, zoonosis

## Abstract

Bovine tuberculosis (BTB) remains a significant zoonotic disease in livestock production, causing considerable animal welfare and economic losses, particularly in communal farming communities where human–animal contact is close, and veterinary services are limited. This study aimed to assess communal cattle owners’ knowledge, attitudes and practices regarding BTB using a structured questionnaire in the Eastern Cape province of South Africa. A cross‐sectional survey design was employed, and a systematic random sampling approach was used to recruit participants from villages in the Makana, Raymond Mhlaba and Enoch Mgijima local municipalities. Face‐to‐face interviews were conducted with 186 cattle owners using a structured questionnaire administered in IsiXhosa or English. Data were analysed using descriptive statistics, Pearson's correlation analyses, and binary logistic regression in SPSS (version 28) and RStudio (version 4.4.2). The findings of this study revealed low awareness: Only 18.8% of cattle owners had heard of BTB, and a limited understanding of its transmission routes, including the consumption of raw milk, undercooked meat and shared grazing land. Although 61.3% of cattle owners supported culling infected cattle, resistance to control measures persists, particularly among experienced farmers, as indicated by a negative Pearson's correlation between experience and attitude towards BTB control (*r* = −0.170, *p* < 0.05). The low awareness levels, insufficient testing and inadequate preventive measures increase the likelihood of undetected infections and contribute to the potential spread of BTB within herds and to human beings.

## Graphical abstract

Bovine tuberculosis remains a major livestock and zoonotic threat in South Africa. This study assessed knowledge, attitudes and practices among communal cattle farmers in the Eastern Cape. Findings revealed low awareness, poor preventive practices, minimal testing and resistance to control measures, underscoring the need for targeted education, surveillance and coordinated control interventions.

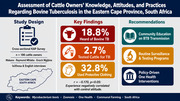

## 1 Introduction

The Eastern Cape province has the largest livestock population in South Africa, with about 3.33 million cattle, which account for approximately 24% of the national cattle herd [1]. Notably, about 78% of these cattle are reared within the communal farming sector compared to the commercial farming sector [2]. Despite this, the province remains vulnerable to Bovine tuberculosis (BTB), a disease that compromises animal health and causes economic losses, particularly through export restrictions. This risk is underscored by research revealing a BTB prevalence rate of 12% in slaughtered cattle in the Eastern Cape province [3].

BTB is caused by *Mycobacterium bovis*, a pathogen that affects cattle and contributes to human tuberculosis cases [4]. The disease remains prevalent in developing nations, with *M. bovis* accounting for roughly 5%–10% of tuberculosis cases and up to 30% of juvenile TB epidemics [5]. In South Africa, the identification of *M. bovis* spoligotypes SB0130 and SB1474 in four sputum samples, where SB0130 had previously been detected in local cattle and wildlife, and SB1474 had been found solely in African buffaloes in an adjacent park, suggests a potential zoonotic transmission pathway [6]. This is particularly alarming as South Africa is among the nations with the highest tuberculosis burden globally, further complicated by the emergence of multidrug‐resistant TB strains [7]. Moreover, a World Health Organisation (WHO) report highlighted South Africa as the only country where TB incidence continued to rise [8].

In most countries, zoonotic tuberculosis transmission to humans is primarily linked to the consumption of contaminated animal‐derived products, especially unpasteurised milk. In contrast, direct transmission through contact with infected animals or between humans occurs less frequently [9]. Additionally, regions where BTB is endemic often coincide with areas of high HIV prevalence, particularly in parts of Africa [10]. South Africa is particularly vulnerable to this dual burden, given its high HIV/AIDS prevalence and the close proximity of livestock and human populations in communal and peri‐urban settings.

To mitigate the spread of BTB, South Africa has implemented a test‐and‐slaughter policy; however, various challenges, such as funding constraints, have hindered its implementation [11]. Community engagement is pivotal in controlling tuberculosis, necessitating a thorough understanding of BTB prevention and control among those occupationally exposed to the disease [12]. Therefore, this study aimed to assess knowledge, attitudes and practices regarding BTB among cattle farmers in the Eastern Cape province of South Africa. The results identify critical knowledge gaps, risky practices and attitudes that may hinder effective BTB control. Through policy formulations, the findings will contribute to improved livestock health management and better protection of farming communities against zoonotic diseases.

## 2 Methods and Materials

### 2.1 Ethical Consideration

Before the study was conducted, ethical clearance (JAJ011SMAG01) was obtained from the University of Fort Hare Inter‐Faculty Human Research Ethics Committee. Informed consent was obtained from each farmer before participating in the research.

### 2.2 Study Site

**FIGURE 1:**
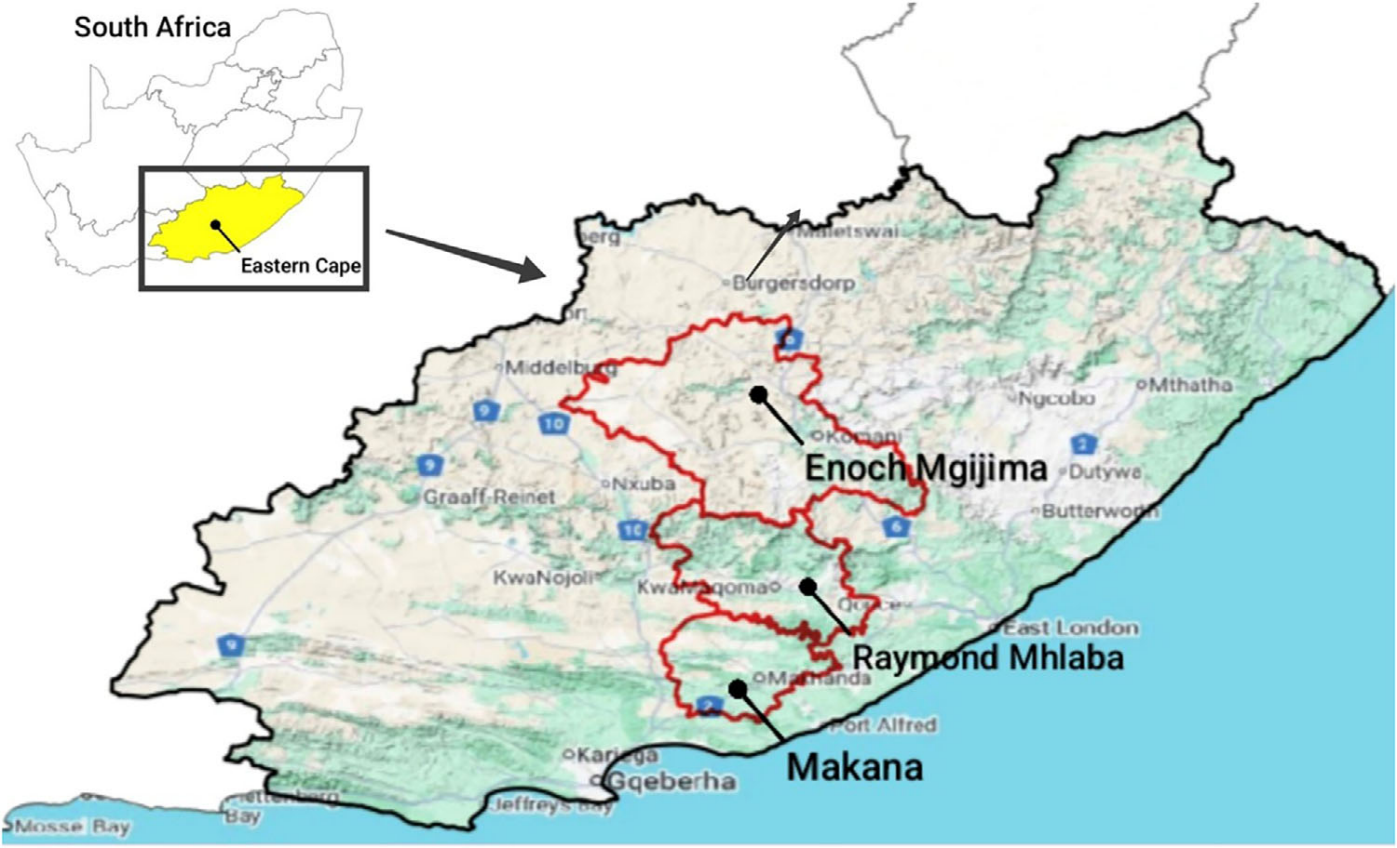
The map shows the study site with the included local municipalities highlighted in red.

This study was conducted in the Eastern Cape province of South Africa, focusing on three local municipalities: Raymond Mhlaba (32.7766° S, 26.6370° E), Makana (33.2415° S, 26.3249° E) and Enoch Mgijima (31.719° S, 26.319° E) as shown in Figure 1. These municipalities represent diverse geographical and socio‐economic conditions, ranging from mountainous terrain to semi‐arid grasslands, well‐suited for cattle farming. The region's climate varies from temperate to warm, with seasonal rainfall that influences agricultural practices, including livestock production [13]. Cattle farming is a key economic activity supporting subsistence and small‐scale commercial farmers in these areas.

Communal cattle farming systems in the Eastern Cape are primarily traditional and subsistence‐oriented, with farmers relying on natural pastures for grazing. Cattle are often kept in open grazing areas with minimal veterinary intervention [14]. The communal nature of land use means that cattle from different households frequently mix, increasing the risk of disease transmission [11]. Farmers in these areas typically have limited access to veterinary services and disease control programs, worsening livestock health challenges. BTB is a significant concern in these systems due to the close interaction between cattle and humans, creating a complex epidemiological landscape.

BTB remains a concern in the Eastern Cape, with incidences reported in communal and commercial farming systems [15]. Studies and veterinary reports have indicated the presence of *M. bovis* in cattle populations, posing a risk to both animal and human health. Inadequate biosecurity measures and the lack of routine BTB testing contribute to the persistence of the disease [16].

### 2.3 Study Design

This study employed a cross‐sectional survey design to assess the knowledge, attitudes and practices of communal cattle owners regarding BTB. A cross‐sectional design was selected because it allows the simultaneous collection of data on multiple variables from a defined population at a single point in time, making it well suited for descriptive and analytical KAP studies. The design was appropriate given the study's dual objectives of describing the distribution of KAP outcomes across the study population and examining associations between demographic characteristics and KAP scores. The study was conducted from August 2024 to August 2025 across three local municipalities in the Eastern Cape province. The unit of analysis was the individual cattle owner, and data were collected through structured face‐to‐face interviews, generating quantitative and self‐reported data.

### 2.4 Sample Size Determination

Yamane's formula was employed to estimate the minimum sample size necessary for the study [17]. The formula is expressed as

$$n=N/(1+N{\left(e\right)}^{2})$$

where *n* is the required sample size, *N* is the total population size (estimated at 400,000 communal cattle farmers in the Eastern Cape), and *e* is the margin of error (0.05).

Using this formula, the calculated minimum sample size was 400 participants. However, due to accessibility and logistical constraints, only 186 cattle farmers participated in the study. This reduced sample size may have lowered the statistical power to detect small or moderate associations, increasing the likelihood of Type II error. Nevertheless, the sample size was sufficient to provide meaningful descriptive insights and to identify key trends in knowledge, attitudes and practices related to BTB among cattle farmers in the study area.

### 2.5 Sampling Method

A systematic random sampling approach was employed to recruit study participants across the selected villages in Makana, Raymond Mhlaba and Enoch Mgijima local municipalities. Systematic random sampling was chosen for three reasons: (i) A complete, pre‐existing register of all communal cattle owners per village was not available, rendering simple random sampling operationally impractical in this resource‐limited field setting; (ii) the method ensured an even geographic spread of selected participants across the full spatial extent of each village, minimising the risk of spatial clustering bias that could arise from convenience‐based recruitment; and (iii) by incorporating a random starting point alongside a fixed sampling interval, the method retained a probabilistic element while remaining feasible under field conditions.

Data collection was conducted through homestead visits along defined village routes. Within each village, the field researcher followed a consistent spatial route from one end of the village to the other, encountering cattle owners sequentially along homestead lines. This directional, route‐based sequence constituted the ordered sampling framework, functioning equivalently to a numbered sampling list in the absence of a formal register.

A sampling interval of *k* = 5 was applied, meaning every fifth cattle owner encountered along the route was invited to participate. The first participant was identified through a random start: Upon arrival at each site, the field researcher numbered the initial group of cattle owners present and randomly selected a number between one and five to designate the first recruit, after which every fifth subsequent individual was approached. Where the accessible population at a site was insufficient to sustain the sampling interval to the required target, the snowball technique was additionally employed to identify and reach further eligible participants within the same village.

### 2.6 Data Collection

Face‐to‐face interviews with a structured questionnaire were conducted at farmers’ homesteads, with each interview lasting approximately 30 min. The questionnaire surveys were conducted in the farmers’ local language (IsiXhosa), except for a few who preferred English.

The questionnaire consisted of four sections: demographic information, knowledge, attitude and practice questions, in both IsiXhosa and English. Demographic information, including education status, sex, race and age, was recorded for each participant. The knowledge part of the questionnaire focused on the farmers’ awareness of BTB, its transmission pathways and prevention and control measures. The attitude section of the survey aimed to determine farmers’ perceptions of the disease's severity, their perceptions of specific risk factors and their control strategies. The practice section of the survey focused on farmers’ practices regarding general hygiene when handling animals, BTB testing of cattle, and the prevention and control of zoonotic diseases. The questionnaire was benchmarked against previously published research, and a pilot study with 10 participants was conducted before the commencement of the main study [12, 18].

### 2.7 Data Management and Statistical Analysis

The dataset was captured on SPSS (version 28, 2021), a statistical software package, to perform descriptive statistical analyses. For evaluation, a scoring system (KAP scores) was implemented, with scores ranging from 0% to 59% categorised as indicative of poor knowledge, and scores between 60% and 100% as reflecting a good level of knowledge, attitude or practice. This was benchmarked using similar previous studies [19]. A good attitude involves the farmer recognising BTB's seriousness, valuing early detection and control measures and being willing to cooperate with veterinary and public health recommendations fully.

In SPSS, frequencies and percentages for all survey questions were calculated to provide a comprehensive overview of the data. RStudio (Version 4.4.2) was also used to assess participants’ knowledge, attitudes and practices. Participants’ scores were calculated for each questionnaire section. In addition to descriptive statistics, inferential statistical methods were used to examine relationships and associations among variables. Binary logistic regression was performed to examine the association between each demographic characteristic and the odds of achieving a good knowledge, attitude or practice score, with results expressed as odds ratios (ORs) and 95% confidence intervals (CIs). Each association was deemed statistically significant if the *p* value was less than or equal to 0.05 (*p* ≤ 0.05). Furthermore, Pearson's correlation test was applied as a two‐tailed analysis to determine the strength and direction of relationships between variables.

## 3 Results

### 3.1 Cattle Owners’ Demographic Characteristics

Communal cattle owners from Enoch Mgijima (44.1%), Makana (30.1%) and Raymond Mhlaba (25.8%) local municipalities were included in the study. As shown in Table 1, the gender distribution of all the respondents was 62.4% male and 37.6% female. The age distribution shows that the majority of farmers were aged 46–60 (37.1%), followed by 31–45 (26.3%) and 60+ (26.9%), whereas the smallest group was 18–30 (9.7%). In terms of race, 95.7% of the participants were black, and 4.3% were coloured.

**TABLE 1:** Demographic characteristics of cattle owners (*n* = 186) in the Eastern Cape province.

		Frequency	Percentage
Municipality	Raymond Mhlaba	48	25.8
	Enoch Mgijima	82	44.1
	Makana	56	30.1
Gender	Male	116	62.4
	Female	70	37.6
Age	18–30	18	9.7
	31–45	49	26.3
	46–60	69	37.1
	>60	50	26.9
Race	Black	178	95.7
	Coloured	8	4.3
	White	0	0.0
	Indian	0	0.0
Education	None	6	3.2
	Primary	43	23.1
	Secondary	117	62.9
	Tertiary	20	10.8
Herd size	1–10	62	33.3
	11–20	79	42.5
	20–30	31	16.7
	>31	14	7.5
Experience	<5	30	16.1
	5–10	60	32.3
	10–15	54	29.0
	>15	42	22.6

Education levels varied, with the majority having secondary education (62.9%), followed by primary (23.1%) and tertiary (10.8%), whereas 3.2% had no formal education. Herd size distribution showed that most farmers had between 11 and 20 cattle (42.5%), followed by those with 1–10 cattle (33.3%). Smaller proportions had between 21 and 30 cattle (16.7%), and 7.5% had more than 31 cattle. Respondents’ experience in cattle farming varied: 32.3% had 5–10 years, followed by 10–15 years (29.0%), 22.6% had more than 15 years, whereas 16.1% had less than 5 years.

### 3.2 Cattle Owners’ Knowledge of BTB

The awareness of BTB among cattle owners in the Eastern Cape province was generally low (Table 2). Only 18.8% of respondents reported having heard about BTB, whereas the majority (81.2%) were unaware of the disease. Only 16.7% of the participants knew that persistent coughing was a symptom of BTB in cattle. Similarly, when asked whether humans can be infected with BTB, 17.2% of the farmers answered ‘Yes’, whereas 82.8% stated they did not know. When asked about the possibility of BTB transmission between cattle and humans, only 16.1% acknowledged it could occur, whereas 83.9% admitted they were unaware.

**TABLE 2:** Awareness and knowledge of bovine tuberculosis among cattle owners (*n* = 186).

	Response	Frequency	Percentage
Have you ever heard about BTB?	Yes	35	18.8
	No	151	81.2
Persistent coughing is not a symptom of BTB in cattle	Yes	1	0.5
	No	31	16.7
	I don't know	154	82.8
Human beings can be infected with BTB	Yes	32	17.2
	No	0	0.0
	I don't know	154	82.8
BTB can spread from infected cattle to humans and vice versa	Yes	30	16.1
	No	0	0.0
	I don't know	156	83.9
Human beings can get BTB from cattle through			
1. Drinking raw (unpasteurised) milk	Yes	30	16.1
	No	0	0.0
	I don't know	156	83.9
2. Eating undercooked meat	Yes	27	14.5
	No	3	1.6
	I don't know	156	83.9
3. Eating the brain	Yes	24	12.9
	No	3	1.6
	I don't know	159	85.5
4. Handling the afterbirth when a calf is born	Yes	23	12.4
	No	4	2.2
	I don't know	159	85.5
Infected cattle can NOT spread BTB to other animals	Yes	23	12.4
	No	4	2.2
	I don't know	159	85.5
BTB can spread through the sharing of grazing land between different herds of cattle from different farmers	Yes	24	12.9
	No	2	1.1
	I don't know	160	86.0

Abbreviation: BTB, bovine tuberculosis.

Knowledge about specific transmission routes was also limited. Only 16.1% of respondents recognised that drinking raw (unpasteurised) milk could lead to infection, whereas 83.9% were unsure. Regarding the risk of eating undercooked meat, 14.5% answered ‘Yes’, 1.6% said ‘No’, and 83.9% were uncertain. Overall, 12.9% of the participants claimed that consuming brain can lead to infection, and 85.5% indicated that they did not know. Similarly, 12.4% of farmers believed that handling the afterbirth of a calf could transmit BTB, whereas 2.2% disagreed, and 85.5% did not know.

Farmers’ understanding of BTB transmission among animals was also limited. Only 12.4% believed infected cattle could not spread BTB to other animals, 2.2% disagreed, and 85.5% were uncertain. When asked about the possibility of BTB spreading through shared grazing land, 12.9% acknowledged this risk, 1.1% disagreed, and 86.0% did not know.

### 3.3 Cattle Owners’ Attitude Towards BTB

Most cattle owners (61.3%) believe that BTB‐positive cattle should be killed, whereas 12.9% disagree with this approach, and 25.8% are uncertain. As shown in Table 3, 45.7% of farmers believe that meat from infected animals is not necessarily bad to consume, whereas 42.5% disagree, and 11.8% are unsure. When asked about the severity of BTB in humans, 66.7% of respondents acknowledged that the disease could cause serious illness or death, whereas 17.2% believe it does not, and 16.1% are uncertain. In terms of reporting suspected BTB cases among cattle, 87.1% of farmers believe it is necessary to notify the authorities, whereas 3.8% do not see the need, and 9.1% are unsure.

**TABLE 3:** Cattle farmer's (*n* = 186) attitude towards bovine tuberculosis.

		Frequency	Percentage
Farmers or authorities should kill BTB‐positive cattle	Yes	114	61.3
	No	24	12.9
	I don't know	48	25.8
Meat from infected animals is not necessarily bad meat to consume	Yes	85	45.7
	No	79	42.5
	I don't know	22	11.8
Bovine TB can cause serious illness or death in humans	Yes	124	66.7
	No	32	17.2
	I don't know	30	16.1
There is no need to notify authorities if there is any suspicion of BTB among cattle	No	162	87.1
	Yes	7	3.8
	I don't know	17	9.1
Campaigns and trainings about BTB are not important	No	158	84.9
	Yes	10	5.4
	I don't know	18	9.7
The open disposal of meat waste and condemned carcasses compromises the environment and also leads to the transmission of zoonotic illnesses	Yes	146	78.5
	No	13	7.0
	I don't know	27	14.5

Abbreviation: BTB, bovine tuberculosis.

Regarding the importance of BTB campaigns and training, 84.9% of respondents believe these initiatives are important, whereas 5.4% disagree, and 9.7% are unsure. Additionally, 78.5% of farmers believe that the open disposal of meat waste and condemned carcasses poses environmental and zoonotic disease risks, whereas 7.0% do not share this concern, and 14.5% are unsure.

### 3.4 Cattle Owners’ Practices With Regard to BTB

Table 4 presents the practices of cattle owners regarding hygiene and BTB‐related infectious diseases. The majority of cattle owners (92.5%) reported that their livestock mix and share grazing land with other farmers’ animals, whereas only 7.5% stated that their cattle do not. Regarding hygiene practices, 87.1% of farmers indicated that they wash their hands before and after handling animal body fluids or collecting samples, such as blood and faeces, whereas 12.9% reported that they do not. Overall, 46.8% of farmers reported using protective clothing when handling animal body fluids or collecting samples, whereas 52.7% stated they do not use gloves, and 0.5% did not respond.

**TABLE 4:** Cattle owners’ (*n* = 186) preventive and general practices towards bovine tuberculosis (BTB).

	Response	Frequency	Percentage
Does your cattle mix and share the same grazing land with other farmers’ livestock?	Yes	172	92.5
	No	14	7.5
Do you wash your hands before and after handling animal body fluid or collection of samples such as blood and faeces?	Yes	162	87.1
	No	24	12.9
Do you use protective clothing (gloves) when handling animal body fluids or collecting samples such as blood and faeces?	Yes	87	46.8
	No	98	52.7
	2	1	0.5
Do you wear protective clothing (gloves) when slaughtering cows?	Yes	61	32.8
	No	125	67.2
Do you milk your cows?	Yes	128	68.8
	No	58	31.2
1. If yes, do you drink raw milk?	Yes	76	59.3
	No	52	40.7
2. Do you boil your milk?	Yes	57	44.5
	No	71	55.6
Do you often go for general medical check‐ups?	Yes	72	38.7
	No	114	61.3
Do you quarantine sick animals or livestock arriving from other areas?	Yes	78	41.9
	No	108	58.1
Do you cull infected animals to control animal disease?	Yes	50	26.9
	No	136	73.1
Have you ever participated in any animal disease or zoonosis training?	Yes	38	20.4
	No	148	79.6

When asked about protective clothing, such as gloves and overalls, used during slaughtering, 32.8% of farmers reported wearing protective clothing, whereas 67.2% stated that they do not. A majority of farmers (68.8%) reported milking their cows. Among those who milk their cows, 59.3% reported drinking raw milk, and 40.7% said they prefer it in other forms. Regarding boiling milk, 44.5% of respondents reported boiling it before consumption, whereas 55.6% stated they do not. In terms of personal health monitoring, 38.7% of farmers reported that they often go for general medical check‐ups, whereas 61.3% do not. When asked about quarantine practices, 41.9% of respondents stated that they quarantine sick animals or livestock arriving from other areas, whereas 58.1% reported not implementing quarantine measures. Farmers were also asked about culling infected animals to control disease, with 26.9% reporting culling and 73.1% stating they do not. Participation in animal disease or zoonosis training was reported by 20.4% of farmers, whereas 79.6% stated that they had never attended such training.

### 3.5 Cattle Owners’ Practices About BTB Testing

Table 5 shows the frequency of BTB testing of cattle herds. Most cattle owners (97.3%) reported that their cattle are never tested for BTB, whereas 1.6% indicated that testing is arranged annually through private veterinarians, and 1.1% reported irregular testing outside of government surveillance programs. Similarly, government veterinary authorities rarely conduct BTB testing among farmers’ cattle: 98.4% of respondents reported that authorities have never tested their livestock, whereas 1.1% reported annual testing, and 0.5% noted irregular testing. Regarding BTB‐related illness or loss among cattle, 97.8% of farmers stated that they had never experienced such cases, whereas 2.2% reported encountering BTB‐related death amongst their cattle. When asked about TB diagnosis within their families, 95.7% of respondents reported no known cases, whereas 4.3% stated that at least one family member had been diagnosed with TB. In terms of cattle being condemned at abattoirs due to BTB, 98.4% of respondents reported that they had never experienced such an issue, whereas 1.6% indicated that they had animals condemned due to BTB.

**TABLE 5:** Cattle owners’ (*n* = 186) bovine tuberculosis (BTB) testing and incidences of the disease.

		Frequency	Percentage
How often do you test your cattle for BTB at your own cost through private veterinarians?	Once a year	3	1.6
	Irregularly	2	1.1
	Never	181	97.3
Do government veterinary authorities ever conduct BTB testing amongst your cattle?	Once a year	2	1.1
	Irregularly	1	0.5
	Never	183	98.4
Have you ever had any BTB‐related illness or loss amongst your cattle?	No	182	97.8
	Yes	4	2.2
Has anyone ever been diagnosed with TB in your family?	No	178	95.7
	Yes	8	4.3
Have any of your animals been condemned at the abattoirs due to BTB?	Yes	3	1.6
	No	183	98.4

### 3.6 Association Between KAP Scores and Cattle Owners’ Demographic Characteristics (Binary Logistic Regression)

**FIGURE 2:**
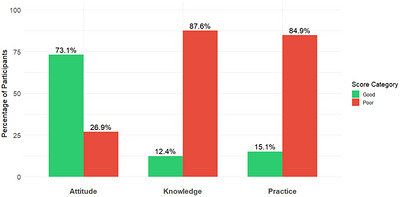
Performance of farmers by KAP score level. Key:Poor = 0–59%score, Good= 60–100% score.

Table 6 presents the binary logistic regression analysis examining the association between cattle owners’ demographic characteristics and their odds of achieving good knowledge, attitude and practice scores regarding BTB. Overall, good knowledge scores were recorded in 24 farmers (12.9%), good attitude scores in 136 farmers (73.1%), and good practice scores in 28 farmers (15.1%) as shown in Figure 2. Regarding knowledge, no statistically significant associations were identified across the demographic variables examined, including municipality, gender, age, race, education level, herd size and farming experience. Although farmers with no formal education showed higher odds of good knowledge compared to those with primary education (OR = 3.800; 95% CI: 0.548–26.360), and farmers aged over 60 years showed higher odds relative to the 18–30 age reference group (OR = 2.526; 95% CI: 0.507–12.600), these associations did not reach statistical significance (*p* > 0.05). Similarly, female farmers showed lower odds of good knowledge compared to males (OR = 0.393; 95% CI: 0.140–1.104), though this trend also fell short of significance (*p* = 0.075).

**TABLE 6:** Pearson correlations between cattle owners’ demographic characteristics and KAP scores.

Municipality
Gender	−0.061
Age	−0.113	0.078
Race	−0.152^a^	−0.001	0.036
Education	0.055	0.062	−0.388^b^	−0.039
Herd size	0.184^a^	−0.046	−0.048	0.031	−0.092
Experience	0.019	0.072	0.496^b^	0.055	−0.195^b^	−0.107
Knowledge	−0.008	−0.107	0.106	0.033	0.061	−0.004	0.121
Practice	−0.009	0.017	0.142	0.010	−0.118	0.084	−0.046	0.107
Attitude	0.165^a^	−0.062	−0.038	−0.015	0.065	0.058	−0.170^a^	0.128	0.127
	Municipality	Gender	Age	Race	Education	Herd size	Experience	Knowledge	Practice	Attitude

^a^Correlation is significant at the 0.05 level (yellow) (2‐tailed).

^b^Correlation is significant at the 0.01 level (orange) (2‐tailed).

Regarding attitude, statistically significant associations were observed for age. Farmers aged 31–45 years showed significantly lower odds of a good attitude towards BTB compared to the 18–30 reference group (OR = 0.133; 95% CI: 0.016–1.096; *p* = 0.050), and this pattern was also significant among farmers aged 46–60 years (OR = 0.126; 95% CI: 0.016–1.005; *p* = 0.033). These findings suggest that younger farmers held more favourable attitudes towards BTB control measures than their middle‐aged counterparts. No significant associations with attitude were found for municipality, gender, race, education, herd size or farming experience.

For practice scores, no statistically significant associations were identified across any demographic variable, although a trend towards significance was noted for Makana municipality relative to Raymond Mhlaba (OR = 2.867; 95% CI: 0.948–8.665; *p* = 0.075) and for farmers owning more than 31 cattle relative to the 1–10 reference group (OR = 3.272; 95% CI: 0.890–12.025; *p* = 0.119). These trends, while not statistically significant, suggest that geographical location and herd size may warrant further investigation in larger samples.

Overall, the logistic regression results indicate that demographic factors were largely not significantly associated with knowledge and practice scores. At the same time, age emerged as a notable predictor of attitude, with younger farmers demonstrating more positive orientations towards BTB control.

### 3.7 Correlation Between Cattle Owners’ Demographic Characteristics and KAP

The correlation analysis between cattle owners’ demographic characteristics, and their KAP scores revealed several significant relationships, as shown in Table 7. The municipality shows a moderate positive correlation with herd size (*r* = 0.184, *p* < 0.05) and attitude (*r* = 0.165, *p* < 0.05), suggesting that farmers’ location may be associated with their herd size and attitudes towards BTB.

**TABLE 7:** Association (binary logistic regression) between cattle owners demographic characteristics and KAP scores.

			Good knowledge (*n* = 24)		Good attitude (*n* = 136)		Good practices (*n* = 28)	
Variable	Category	Freq. (%)	OR (95% CI)	*p* value	OR (95% CI)	*p* value	OR (95% CI)	*p* value
**Municipality**								
	**Raymond Mhlaba (ref)**	9 (18.8)	**Ref**.	na	**Ref**.	na	**Ref**.	na
	Enoch Mgijima	6 (7.3)	0.342 (0.114–1.031)	0.085	0.995 (0.455–2.178)	1.000	1.060 (0.334–3.370)	1.000
	Makana	9 (16.1)	0.830 (0.300–2.294)	0.798	1.510 (0.619–3.682)	0.375	2.867 (0.948–8.665)	0.075
**Gender**								
	**Male (ref)**	19 (16.4)	**Ref**.	na	**Ref**.	na	**Ref**.	na
	Female	5 (7.1)	0.393 (0.140–1.104)	0.075	0.979 (0.502–1.910)	1.000	1.536 (0.683–3.454)	0.300
**Age (years)**								
	**18–30 (ref)**	2 (11.1)	**Ref**.	na	**Ref**.	na	**Ref**.	na
	31–45	6 (12.2)	1.116 (0.204–6.111)	1.000	0.133 (0.016–1.096)	**0.050**	0.698 (0.155–3.144)	0.693
	46–60	4 (5.8)	0.492 (0.083–2.929)	0.599	0.126 (0.016–1.005)	**0.033**	0.948 (0.235–3.835)	1.000
	>60	12 (24.0)	2.526 (0.507–12.600)	0.323	0.186 (0.022–1.550)	0.160	0.952 (0.223–4.068)	1.000
**Race**								
	**Black (ref)**	23 (12.9)	**Ref**.	na	**Ref**.	na	**Ref**.	na
	Coloured	1 (12.5)	0.963 (0.113–8.188)	1.000	0.598 (0.138–2.600)	0.445	— (—)	—
**Education**								
	**Primary (ref)**	5 (11.6)	**Ref**.	na	**Ref**.	na	**Ref**.	na
	None	2 (33.3)	3.800 (0.548–26.360)	0.199	— (—)	—	1.576 (0.167–14.891)	1.000
	Secondary	13 (11.1)	0.953 (0.314–2.893)	1.000	1.228 (0.584–2.580)	0.588	0.598 (0.209–1.714)	0.338
	Tertiary	4 (20.0)	1.900 (0.427–8.459)	0.596	2.806 (0.700–11.254)	0.220	0.422 (0.073–2.438)	0.421
**Herd size (cattle)**								
	**1–10 (ref)**	7 (11.3)	**Ref**.	na	**Ref**.	na	**Ref**.	na
	11–20	10 (12.7)	1.139 (0.407–3.186)	1.000	1.060 (0.507–2.214)	1.000	0.853 (0.324–2.249)	0.807
	20–30	6 (19.4)	1.886 (0.575–6.189)	0.347	1.705 (0.599–4.853)	0.451	0.872 (0.246–3.093)	1.000
	>31	1 (7.1)	0.604 (0.068–5.351)	1.000	1.023 (0.284–3.688)	1.000	3.272 (0.890–12.025)	0.119
**Experience (years)**								
	**<5 (ref)**	4 (13.3)	**Ref**.	na	**Ref**.	na	**Ref**.	na
	5–10	5 (8.3)	0.591 (0.146–2.385)	0.474	0.750 (0.258–2.183)	0.792	0.858 (0.230–3.198)	1.000
	10–15	6 (11.1)	0.812 (0.210–3.141)	0.740	0.594 (0.204–1.728)	0.440	2.061 (0.606–7.007)	0.274
	>15	9 (21.4)	1.773 (0.490–6.408)	0.537	0.558 (0.184–1.689)	0.417	0.684 (0.157–2.985)	0.711

Abbreviations: CI, confidence interval; OR, odds ratio.

Age is positively correlated with experience (*r* = 0.496, *p* < 0.01), indicating that older farmers generally have more years of farming experience. However, education is negatively correlated with experience (*r* = −0.195, *p* < 0.01), suggesting that more experienced farmers tend to have lower levels of formal education. Additionally, experience is negatively correlated with attitude (*r* = −0.170, *p* < 0.05), suggesting that more experienced farmers may have less favourable attitudes towards BTB‐related practices. Knowledge, practice and attitude show limited correlations with demographic variables, with no significant relationships observed between knowledge and any demographic factor. Similarly, practice shows only weak correlations with most characteristics, although a minor positive correlation with attitude (*r* = 0.127) is observed. Overall, the findings suggest that although certain demographic factors influence attitudes and practices, knowledge remains relatively unaffected by these variables.

## 4 Discussion

The study reveals a low level of awareness of BTB among communal cattle owners, consistent with a study in Zambia that reported that only 39.6% of cattle owners had heard of the disease [20]. Findings confirm that inadequate awareness of BTB risk factors is not unique to the Eastern Cape [12]. The majority of cattle owners in the present study did not recognize persistent coughing as a major symptom of the disease. This gap risks delaying detection and impeding the timely implementation of control measures [21].

Knowledge about transmission routes, including raw milk consumption, undercooked meat and shared grazing land, was similarly poor among the study participants. This is a critical concern, given that zoonotic tuberculosis primarily transmits through contaminated animal products, particularly unpasteurised dairy [9], and that inadequate farmer awareness of zoonotic diseases contributes to continued transmission and outbreaks in affected communities [22]. These findings carry particular weight within the broader South African context, where tuberculosis remains a major public health challenge and poor BTB awareness among cattle owners adds a substantial zoonotic risk in communities that experience close human and animal contact alongside limited access to veterinary and public health services [10, 11].

The study identified critical gaps in the preventive and control practices of cattle owners. The large majority reported allowing unrestricted livestock mixing, a practice that studies consistently identify as a significant risk factor for BTB transmission [23, 24], whereas fewer than half applied quarantine measures, further compounding transmission risks within and between herds [25]. Most cattle owners did not wear protective clothing during slaughter, despite strong evidence that such measures substantially reduce the risk of direct BTB infection [26].

A substantial proportion of cattle owners held the view that meat from infected animals is not necessarily harmful to consume, reflecting a gap in understanding of foodborne zoonotic disease risks that has been documented in similar farming communities across the region [10, 11, 16]. BTB testing was virtually absent across the surveyed herds. Kgasi and Michel [27] showed that farmers tend to rely on the government for disease control, and the absence of compensation in the event of culling, alongside the lack of government‐subsidised testing programmes, further deters compliance [11, 28, 29, 30]. Without adequate testing, infected animals remain undetected, enabling silent transmission within and across farming communities [31].

Attitudes towards BTB control were generally favourable, with the majority of cattle owners supporting the culling of infected animals and recognising the importance of reporting suspected cases to the relevant authorities. However, resistance to culling persists in communities where cattle hold significant economic and cultural value, thereby limiting the real‐world effectiveness of the test‐and‐slaughter policy [32]. The generally positive attitude scores stand in notable contrast to the poor knowledge and practice scores recorded across the study population, pointing to a deeper structural issue in how awareness translates into action.

A concerning disconnect between attitude and practice emerged throughout the study. Favourable attitudes did not translate into adequate implementation of preventive measures, a pattern well documented in zoonotic disease management and reflecting the complex relationship between awareness, intention and behaviour [33]. This attitude–practice gap highlights that education alone is insufficient; structural support, in the form of government‐subsidised testing, accessible veterinary services and community‐based outreach programmes, is equally necessary to drive meaningful and sustained behavioural change among communal cattle owners.

The binary logistic regression analysis indicated that demographic characteristics were largely not significant predictors of knowledge and practice scores among cattle owners. This finding resonates with similar studies in sub‐Saharan African farming communities, where demographic variables, such as sex, age, education and experience, consistently failed to predict levels of knowledge about BTB [34, 35]. The absence of strong demographic predictors suggests that BTB awareness gaps in communal farming communities are more likely driven by systemic deficiencies, including limited access to veterinary extension services, inadequate public health education and historically weak disease surveillance, than by individual characteristics. The correlation analysis further revealed that municipality significantly associated with both herd size and attitude, whereas farming experience correlated negatively with attitude towards disease control, an association consistent with evidence that long‐standing farming practices can entrench resistance to veterinary recommendations [36, 37]. These selective but meaningful associations reinforce the value of location‐sensitive interventions even in settings where demographic factors do not broadly predict KAP outcomes.

The cumulative findings of this study point clearly to the need for a coordinated, multi‐sectoral response to BTB in communal farming areas of the Eastern Cape. A One Health approach that brings together communal cattle owners, veterinary services, public health authorities and government agencies offers the most credible pathway to reducing the disease burden and preventing zoonotic transmission. Such an approach must address the structural barriers that perpetuate poor knowledge and practice, including the absence of routine herd surveillance, limited veterinary outreach, inadequate compensation frameworks, and the long‐standing divide between animal and human health messaging that has obscured the zoonotic implications of BTB in this setting [38]. Without this integrated commitment, the gap between attitude and action will persist, and BTB will continue to pose an unacceptable risk to cattle, farming livelihoods and human health in the Eastern Cape and beyond.

## 5 Conclusion

This study demonstrates critical gaps in the knowledge, attitudes and practices of communal cattle owners in the Eastern Cape province regarding BTB. Most cattle owners were unaware of the disease, its zoonotic potential, key symptoms and primary transmission routes. Although attitudes towards disease control were generally positive, the persistent disconnect between attitude and practice remains a significant barrier to effective BTB management in communal farming settings. The findings underscore the urgent need for targeted education programmes, government‐led testing initiatives and integrated surveillance strategies that address the livestock, human and environmental interface. A One Health approach that actively involves communal cattle owners, veterinary services and public health authorities is essential to reducing the burden of BTB and preventing zoonotic transmission in the Eastern Cape and similar settings across South Africa.

## 6 Limitations of the Study

The achieved sample size (*n* = 186) was lower than the calculated sample size (*n* = 400), potentially reducing statistical power and limiting the generalisability of the findings.

The cross‐sectional design limits causal inference about the associations between socio‐demographic factors and KAP outcomes.

Reliance on self‐reported data may have introduced recall and social desirability bias.

The absence of laboratory confirmation of BTB in cattle herds prevented direct linkage between reported practices and actual disease status.

## Author Contributions

**Effort Maguta**: conceptualisation, writing, data analysis, methodology. **Anita Michel**: supervising, funding acquisition, conceptualisation, methodology, editing, data analysis. **Ishmael Festus Jaja**: supervising, funding acquisition, conceptualisation, methodology, editing, data analysis. All authors agree to be accountable for all aspects of the work.

## Funding

This study was supported by the Belgian Directorate‐General for Development Cooperation (DGD) through the DGD–Institute of Tropical Medicine (ITM) Framework Agreement 5 covering the period 2022 to 2026.

## Conflicts of Interest

The authors declare no conflicts of interest.

## Data Availability

The data that support the findings of this study are available from the authors upon request.
